# Revealing the Causes of Dyslexia through a Differential Diagnosis, a Short-Term Effective Treatment and an Appropriate Conceptual Framework

**DOI:** 10.3390/diagnostics14171965

**Published:** 2024-09-06

**Authors:** Reinhard Werth

**Affiliations:** Institute for Social Pediatrics and Adolescent Medicine, Ludwig-Maximilians-University of Munich, Haydnstr. 5, D-80336 Munich, Germany; r.werth@lrz.uni-muenchen.de; Tel.: +49-(0)1733550232; Fax: +49-308337940

**Keywords:** dyslexia, homonymous hemianopia, reading impairment, reading therapy, causes, conditions

## Abstract

Various different impairments and their interactions can cause reading problems referred to as “dyslexia”. Since reading requires the interaction of many abilities, the impairment of each of these abilities can result in dyslexia. Therefore, the diagnosis must differentiate various kinds of dyslexia. The diagnosis of a certain kind of dyslexia cannot be delimited to the investigation and description of symptoms but must also include the investigation of the causes of each kind of dyslexia. For this purpose, a scientifically unequivocal concept of causation and appropriate methods are needed to distinguish them from co-existing impairments that have no causal influence on reading performance. The results of applying these methods cannot be adequately accounted for by a non-scientific, intuitive understanding of necessary and sufficient conditions and causation. The methods suitable for revealing the causes of dyslexia are described in detail, and the results of applying these methods in experiments, in which 356 children with developmental dyslexia participated, are reviewed. Since the concepts of “necessary” and “sufficient” conditions and “causation” proposed in the philosophy of science are not suitable for describing causes of dyslexia and their interaction, they are replaced by a more detailed, experimentally based conceptual framework that provides an accurate description of the conditions required for correct reading and the causes of dyslexia.

## 1. Introduction

Reading problems termed “dyslexia” may be caused by a variety of impairments, such as visual or auditory impairments, impairments in processing sensory input, attention deficits, mental disorders, or insufficient schooling. Which kind of dyslexia is diagnosed depends on the impairments that cause the reading problems. Hemianopic dyslexia can, for instance, only be diagnosed if it is confirmed that it is exclusively caused by a homonymous hemianopia. Neglect dyslexia presupposes that reading problems are exclusively due to visual neglect. In contrast, developmental dyslexia (DD) is not defined with respect to its causes; the diagnosis only requires that certain causes are ruled out. In the Diagnostic and Statistical Manual (DSM 5) [1], DD is regarded as a specific learning disorder that is indicated by “… inaccurate and effortful word reading, … difficulty understanding the meaning of what is read …” and “… difficulty with spelling”. These difficulties must have persisted for at least six months and remain below the skills expected for the chronological age. The difficulties “… are not better accounted for by intellectual disabilities, uncorrected visual or auditory acuity, other mental or neurological disorders, psychological adversity, lack in the proficiency in the language of academic instruction, or inadequate educational instruction …” (DSM 5 2013, p. 67). According to these criteria, approximately 5–15% of school children in the USA are dyslexic [2,3,4]. In Germany, the proportion of fourth graders with dyslexia is also estimated at between 15% [5] and 25% [6].

However, such a dyslexia diagnosis rests on a vaguely described list of excluded impairments lacking unequivocal diagnostic criteria. If reading problems are not caused by an impairment of the refractive media of the eye or a retinal illness, an impairment of the auditory system, motor impairment of eye movements, “… intellectual disabilities, mental disorders, psychological adversity, lack in the proficiency in the language of academic instruction, or inadequate educational instruction…” [1], the reading problems must be caused by an impairment of brain functions, which the definition does not specify. The fact that reading requires many abilities and that an impairment of at least one of these abilities can cause reading problems referred to as dyslexia shows that there are various kinds of dyslexia. DD is a summary of a number of reading problems whose causes are unknown, excluding causes that are vaguely accounted for in the Diagnostic and Statistical Manual (DSM).

In order to read flawlessly and fluently, the reader must fixate on the right location within a word and several letters that constitute a word or word segment must be recognized simultaneously. To achieve this, the gaze must be focused for a sufficiently long time on the word or word segment to be read. After the end of the fixation time that is needed to recognize the word or word segment, saccades in the reading direction, whose amplitudes do not exceed the length of the sequence of letters that can be recognized simultaneously must be programmed and executed. The correct phonemes must be retrieved from memory, words or word segments must be stored in memory and composed into sentences, and meaning must be given to the words and sentences. When reading aloud, the reader must not start pronouncing the word or word segment before the corresponding sound sequence has been completely retrieved from memory. The diagnosis must distinguish between the various kinds of dyslexia that are caused by the impairment of different abilities. The different types of dyslexia can only be differentiated from one another if the diagnosis relates to their different causes. Therefore, revealing the different causes of reading impairments constitutes an essential element of the diagnosis. Conventional reading tests [7] indicate whether a child’s reading ability is below that expected for his or her chronological age. However, these tests do not reveal the different causes of reading disturbances and cannot distinguish between the different kinds of dyslexia. As a result, reading therapies cannot be tailored to the different causes of dyslexia and are unspecific, long-term, and of limited success.

Although the knowledge of the causes of reading problems is a presupposition for a sufficiently detailed diagnostic, discussions about the causes of dyslexia have hitherto rested only on an intuitive concept of “causation”. The question of how to specify a scientific concept of cause and which experimental methods are suitable for distinguishing causes from non-causal relationships has not been addressed in the research on dyslexia, and previous attempts in the philosophy of science to clarify these concepts [8,9,10,11,12,13,14,15,16,17] have been ignored. This resulted in an unclear distinction between the causes of dyslexia and concomitant impairments without a causal relationship to dyslexia [18] and in speculative assertions about the causes of dyslexia. It has been hypothesized that developmental dyslexia may be due to an unusual masking (crowding) effect in the visual field [19,20,21,22,23,24,25,26,27,28,29,30], an insufficient ability to expand the visual field of attention [31,32,33,34,35,36,37,38,39,40], an impairment in discriminating auditory stimuli [41,42,43,44,45], a lack of eye movement control during reading [46,47,48,49,50,51,52,53,54,55,56,57,58,59,60,61,62,63,64], and impaired phonological awareness [65,66,67,68,69,70,71,72,73,74,75]. Phonological awareness includes various abilities, such as splitting words into syllables and sounds [65,66,67,68,69,70,71]; identifying phonemes in words [68,72,73]; naming letters, objects, numbers, and colors [68,74]; and rhyming [68,69,70,71,72,73,74,75]. The phonological awareness theory suggests that dyslexia is the result of an inability to associate a visually recognized sequence of letters that make up words with the appropriate sequence of sounds. However, these impairments can only be considered concomitant impairments because they have not been proven to cause dyslexia [18]. Experimental methods that are based on a clear concept of “cause” and that can reveal the causes of DD show that these assumptions about the causes of DD are not justified [18,76,77,78,79,80,81,82]. Without unequivocal criteria informing us what necessary and/or sufficient conditions and causal relationships are and which methods can distinguish between causes and concomitant impairments without a causal influence, assertions about the causes of dyslexia are only hypotheses without a scientific background.

Since the concepts “causation”, necessary conditions”, and “sufficient conditions” proposed in the philosophy of science cannot adequately describe the various conditions and their interactions that cause dyslexia, a more sophisticated conceptual framework is required.

The aim of this present study is to describe the experimental methods applied to identify the causes of dyslexia in detail, to propose a suitable conceptual framework that is different from earlier concepts advocated in the philosophy of science [8,9,10,11,12,13,14,15,16,17], and to demonstrate the application of these methods and the conceptual framework on reading problems. It will be shown that the causes of reading problems may differ among children and that the therapy must take these differences into account [76,77,78,79,80]. The studies demonstrate that knowledge of the causes of dyslexia enables a detailed diagnosis of reading problems and a therapy tailored to each child’s reading problem, resulting in an immediate improvement in children’s reading performance.

## 2. How to Reveal the Causes of Dyslexia

### 2.1. The Example of Hemianopic Dyslexia

Whereas the investigation of the causes of many kinds of reading problems termed DD is complex and requires sophisticated methods, the causal influence of HH on reading problems can be revealed rather easily by systematically eliminating or compensating alleged causes during the diagnostic process. Hemianopic dyslexia cannot yet be diagnosed when visual perimetry identifies a homonymous visual field defect, such as a complete HH, homonymous scotomata involving foveal or perifoveal areas, an increased foveal and perifoveal luminance-difference threshold, or a cerebrally decreased visual acuity coexisting with reading problems. It must also be demonstrated that the visual field defect is the only possible cause of the reading problem. A cerebral lesion often results in a homonymous visual field defect coexisting with other neurological impairments so the interaction of the HH with these impairments influences the reading ability. In such cases, the diagnosis must distinguish between reading problems caused by HH and the impact of other impairments on the reading capacity.

Patients with complete homonymous hemianopia (HH) that includes the fovea regularly suffer from reading problems known as “hemianopic dyslexia” [83,84,85,86,87,88,89,90,91,92]. When reading, eye movements must shift the word segment into the fovea and parafoveal area, which has a sufficiently high visual acuity. To recognize as many letters as possible, the gaze is directed approximately to the middle of the word segment [93,94]. If the left visual hemifield is blind and if blindness includes the fovea, letters at the beginning of the word segment to be read are located in the blind left foveal region. Then, letters at the beginning of the word segments are not registered. If a patient suffers from right HH, letters in the right half of the word segment are in the blind right visual hemifield. These patients typically leave out the end of the word segments to be read. They also cannot see the location of the target of the saccade that must be executed in the reading direction after a word or word segment has been read because the target of the saccade is in the blind area. To fixate on the next word or word segment to be read, they execute a succession of hypometric saccades to search for the target of the eye movement to fixate on the next word or word segment that they expect in the hemianopic hemifield. The causes of reading problems seem obvious in these patients since it is clear that a person cannot read if they cannot see all the letters in a word to be read. The impact of HH on reading problems can be demonstrated in two ways. The seeing area in the foveal and parafoveal area can be extended by visual field training. Such training has proven to be highly effective in adults and children [95,96,97,98,99,100,101,102,103,104,105,106,107,108,109,110]. It can be concluded that HH had a causal influence on the reading impairment when reading normalizes after successful visual field training and after a sufficient reduction of the blind visual area. 

If the visual field training is not effective, hemianopia can be compensated by a new eye movement strategy. Readers with right homonymous hemianopia including the fovea cannot see the location of the target of the saccade that must be executed in the reading direction after a word or word segment has been read because the target is in the blind area. During the eye movement training, the children must direct their gaze to the end of the word or word segment to be read to compensate for right homonymous hemianopia including foveal and parafoveal regions. The patients can then project more letters in the word or word segment to be read onto the remaining left half of the foveal and parafoveal region. To fixate on the next word or word segment to be read, they must execute one hypermetric saccade in the reading direction followed by a correction saccade opposite to the reading direction aiming at the end of the word or word segment that is now in the good left hemifield. 

In contrast, patients with left HH involving the foveal and parafoveal area must direct their gaze to the beginning of a word to shift the word or word segment to be read into the intact right visual hemifield. The patients can then see the text to the right of the word or word segment that they are reading and can locate the target of the saccade that must be executed to read the next word or word segment. When reading improves significantly after the intact visual area has increased or after adopting an appropriate eye movement strategy, this demonstrates that both the HH and the lack of compensatory eye movements had a causal influence on the origin of hemianopic dyslexia. HH is only a cause for hemianopic dyslexia if the patient does not exert compensatory eye movements, and the lack of compensatory eye movements is a cause for hemianopic dyslexia in the presence of HH.

Patients with homonymous hemianopia suffer from postgeniculate cerebral damage that may also result in hemispatial neglect on the side of the hemianopic visual field defect. Whereas patients with homonymous hemianopia without neglect search for objects in the half of the space to the left and right of their body midline and on both sides of a text, patients with hemispatial neglect do not register the presence of the half of space to the left or right of their body midline [111,112,113,114,115]. These patients do not search for objects in the half of the space to the left or right of their body midline, do not detect objects in the neglected half of space, do not detect objects on one half of a sheet of paper, and do not register the left or right halves of objects. When reading, they typically ignore the left or the right halves of the text. When there is only mild left hemispatial neglect, or when severe left hemispatial neglect has disappeared and only mild symptoms remain, patients may still miss the first word in a text or neglect the beginnings of words.

Such symptoms may still be present after visual field training was successful and a new eye movement strategy has been adopted by the patient. Since a right HH occurs after damage to the left cerebral hemisphere, the lesion may affect brain areas that are involved in understanding speech and speech production, which also impairs reading performance. Therefore, symptoms of reading deficiency that are not caused by HH, a lack of compensatory eye movements, or hemispatial visual neglect may also remain. In these cases, HH is not the only cause for the reading problems, and the diagnosis of hemianopic dyslexia is not justified. This example shows that a correct and precise diagnosis cannot be limited to the description of symptoms but must also include considerations of all causal relationships and their interactions. Since this can only be provided by improving reading ability after ruling out all possible causes, the diagnosis includes a therapeutic approach.

It is only difficult to exclude the causes of reading problems when DD is present with or without other possible causes of a reading disorder if one agrees with the prevailing opinion that the causes of DD are unknown. The following shows that the assumption that the causes of DD are unknown is incorrect and demonstrates which methods can be used to identify the causes of the different types of dyslexia referred to as DD.

As already mentioned above, developmental dyslexia is regarded as poor reading performance that is not caused by a visual impairment such as a visual field defect, reduced visual acuity, a motor eye movement disturbance, an auditory disorder, a neurological or psychiatric disease, or inadequate schooling. The diagnosis of DD is usually based on tests that examine whether the children’s reading capacity is outside the range of normal age-matched readers. Testing the rate of errors when reading single natural words, pronounceable pseudowords, and text, as well as the time needed to complete a reading task, may be sufficient to determine whether a child’s reading ability is within the normal range or by how many standard deviations it deviates from normal. These tests do not tell us why a child´s reading performance is below the age norm. Since the reading process requires many different abilities, different abilities may be impaired in different children. It has been shown [76,77,78,79,80] that different impairments can cause reading problems diagnosed as “dyslexia”. There is not only one kind of dyslexia, and the kinds of dyslexia can vary among children.

Since the goal is to improve the reading ability of children with dyslexia, it is also important to find out what causes the reading disorder in each individual child. Appropriate therapy can be designed that eliminates or compensates for the causes of the reading problems only when the causes of dyslexia are known. 

### 2.2. What Causes Misreading of Words within a Fixation Interval?

Different methods can be used to detect the causes of the inability to read words within a fixation interval. As already discussed above, the cause of hemianopic dyslexia must be confirmed by examining whether the reading disorder improves significantly when the suspected cause is eliminated.

To investigate the causes of different kinds of dyslexia termed DD, it must be demonstrated that reading performance immediately improves in the presence of that feature and deteriorates in its absence, while all other influences on reading performance remain stable. We achieved this by examining reading performance in the presence and absence of various features [76,77,78,79,80]. The features manipulated were the fixation time, the number of letters, and the verbal response time needed for children with dyslexia to read at least 95% of a list of 20 pronounceable pseudowords correctly. We chose pseudowords because familiar natural words can be guessed when only a few letters in the words are recognized. Pseudowords can only be recognized if every letter is recognized. Therefore, it cannot be tested with natural words whether every letter in a word is recognized. The pseudowords we used were composed of letter sequences that also occurred in natural colloquial words and were as easily pronounceable as words used in everyday language. Reading tachystoscopically presented pseudowords is different from reading text. When reading tachystoscopically presented pseudowords, no eye movements need to be planned and executed, no words need to be combined into sentences, and no meaning needs to be attached to the pseudowords. Therefore, all effort can be focused on the recognition of all letters in the pseudowords. The pseudowords can be split at any location and can be divided into segments of different lengths. This is not the case with natural words.

To distinguish between different possible causes of dyslexia, the reading capacity must be tested under conditions that change one possible cause after another, and it must be registered how the change in each condition affects reading capacity. For example, 3-letter pseudowords are presented for 250 ms while fixation is controlled. If three letters are recognized simultaneously, the number of letters can be increased until not all letters are recognized simultaneously. This demonstrates how many letters a child can recognize simultaneously at a fixation time of 250 ms. If three letters are not correctly pronounced, the child should be asked to spell and write the pseudoword. If their response is correct, the child may still be unable to pronounce the pseudoword correctly, although it has been visually recognized. In this case, the time needed to pronounce the word can be prolonged. This can be achieved by presenting a sound, indicating when the child is allowed to start pronouncing the word.

We developed computer software that allowed us to manipulate the presentation time (i.e., fixation time), the number of letters contained in a pseudoword, and the time from fixation onset to the pronunciation of the pseudoword (verbal reaction time). If the children were unable to recognize all the letters, the fixation time was increased in steps of 50 ms up to 500 ms. The number of letters in the pseudowords that each child was able to recognize at each fixation time and the required verbal reaction time were examined. One feature (e.g., the fixation time) was varied while the other features (number of letters in the pseudoword and verbal reaction time) remained constant, or the fixation time and verbal reaction time remained constant and the number of letters was varied. All 200 children who participated in these tests were able to pronounce at least 95% of a sequence of 20 pseudowords correctly when presented with an appropriate fixation time when the number of letters in the pseudowords did not exceed an appropriate number, and when an appropriate verbal reaction time was maintained. Extending the verbal reaction time did not always improve the children’s ability to read pseudowords. In our studies [80,81,82], the fixation time, the number of letters that could be recognized simultaneously, and the time it took to pronounce the word correctly varied from child to child. Some children were even able to recognize up to six letters simultaneously within a fixation time of 500 ms or less. The average verbal reaction time was between 1316 ms (SD = 712 ms) and 1670 ms (SD = 641 ms). The length of the pseudowords that children can recognize and the fixation time required to do so differs considerably among children. Children who can recognize six letters simultaneously within 350 ms but still have reading problems because they do not meet the required verbal reaction time have a different type of dyslexia than children who can only recognize three letters simultaneously within 500 ms.

This shows that children with dyslexia have not lost the ability to retrieve the sound sequence that corresponds to a completely seen sequence of letters that make up a word from memory and that the inability to retrieve the sound sequence that corresponds to a completely seen sequence of letters that make up a word from memory is not the cause of DD, as assumed by the phonological awareness theory [65,66,67,68,69,70,71,72,73,74,75]. The experiments demonstrate that the ability to read all pseudowords correctly can be established, that too short a fixation time and/or trying to recognize more letters simultaneously than the reader can recognize and/or too short a verbal reaction time impair reading performance [78,79,80]. A sufficiently long fixation time and/or not trying to recognize more letters simultaneously than the reader can recognize and/or a sufficiently long verbal reaction time therefore constitute necessary conditions for the correct recognition of pseudowords. These necessary conditions could only be demonstrated by creating conditions under which a person could recognize at least 95% of the pseudowords. This shows that the diagnosis is also based on creating conditions under which sufficient reading ability is established.

In languages with a high grapheme–phoneme correspondence, such as German, Italian, and Spanish, children have no problem pronouncing the words, provided they have learned which sounds correspond to which letters. In languages such as English and French, the same letter can be pronounced differently depending on the letters that precede or follow it, and a phoneme can correspond to different sequences of letters. For example, in French, “o”, “eau”, and “eaux” correspond to a single phoneme. In English, the same phoneme may correspond to “au”, “aw” and “o”. A child may have a reduced ability to store the different pronunciation rules needed to pronounce English or French words correctly in memory and to retrieve the correct phonemes quickly when reading. These memory problems must be separated from the impairments described above, as they may be a component of dyslexia that occurs only in certain languages. This means that the ability to read pseudowords and natural words must be tested under conditions in which the children are familiar with all the letters and have no problem associating graphemes with phonemes. All the children who participated in our studies were able to correctly associate graphemes and phonemes but still had significant reading problems.

### 2.3. Inappropriate Eye Movements Cause Dyslexia

We have hitherto only considered the ability to read pseudowords presented tachystoscopically at a location where the children focused their gaze. However, to be able to read a text, additional conditions must be met. The question of whether eye movements are the cause or consequence of a reading disorder has been the subject of a controversial debate [18,46,47,48,49,50,51,52,53,54,55,56,57,58,59,60,61,62,63,64]. The importance of eye movements for the improvement of reading ability in patients with HH has already been explained in Section 2. Appropriate reading eye movements are also necessary conditions required for correct reading and cannot be replaced by other conditions. When reading, word recognition takes place after a saccade has been completed. During a saccade, word recognition is not possible because visual functions are inhibited. After the saccade is completed, visual functions recover, and the ability to recognize words is restored. Reading errors will occur if the reader does not prolong the fixation interval as long as the visual system needs to recover and recognize the word segment after the saccade.

When reading text, another important source of reading errors is the attempt to simultaneously recognize words or word segments that consist of more characters than the reader can recognize simultaneously. If premature saccades are an irreplaceable sufficient condition for reading failure, then reading should improve if premature saccades are prevented. We developed a computer program that helps children to read only words or word segments that do not contain more letters than they can simultaneously recognize. A yellow fixation mark indicates the point where the gaze should be directed, and a green cursor (segment cursor) indicates the length of the word segment to be read. Each time a segment is recognized, the next segment to be read is displayed. The yellow fixation mark moves to the middle letter of the next word or word segment, indicating the target of the saccade, i.e., where the gaze should be directed to read the next word segment. A green cursor then indicates how many letters in the word segment should be read. The fixation mark and segment cursor move from word segment to word segment as they are read. If this does not sufficiently prevent the child from terminating fixation on a word segment by making a premature saccade before the required fixation time has elapsed, the text to the right of a word segment is not displayed. The next word segment to be read is only displayed if the previous word segment was fixated long enough and recognized correctly. Eye movements are monitored, analyzed, and stored online. The reader is thus forced to fixate segments of a convenient length for at least the time interval required to recognize word segments of that length.

When reading, the child must execute a saccade in the reading direction to shift one word segment after the other into the area of highest visual acuity. Therefore, the text must be divided into word segments that do not contain more letters than the reader can recognize simultaneously. The pseudowords can be split at any location. This is not the case with natural words. In many languages, there are single phonemes that correspond to a sequence of characters such as “ch”, “sch”, “ie”, and “ah” in German; “ea” “au”, and “oa” in English; and “au”, eaux”, and “en” in French. Such a sequence of characters should not be split in reading training. The amplitude of reading saccades is not always the same but must vary according to the requirements of the text. Some children who can, e.g., simultaneously recognize four letters in pseudowords, need not always split the text into exactly four-letter segments. When the fourth letter occurs between a sequence of characters that should not be separated, the segment to be read should be a three-letter segment or even a two-letter segment. It is only important that children do not try to read more letters at a time than they can.

**Figure 1:**
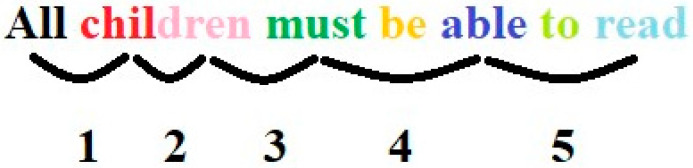
Appropriate and inappropriate reading eye movements. Sequences of letters of different colors indicate different word segments that do not contain more letters than the reader can recognize simultaneously. Arcs below the text indicate saccades from one word segment to the next. Numbers denote successive saccades. The saccades 1–3 are appropriate for a reader who cannot recognize more than four letters simultaneously. One word segment follows the next without a gap. Saccades 4 and 5 exceed the number of letters that the reader can recognize simultaneously. This results in gaps (in which “to” and “be” are located) between the word segments that are not recognized when reading.

To avoid missing any letters, each of these word segments must follow one another without a gap. The amplitudes of the saccades in the reading direction must not exceed the length of the word segments that can be recognized. If the saccades are too large, not all letters in the word segments can be recognized (Figure 1). Since children differ considerably in the length of word segments they can recognize simultaneously, the amplitude of eye movements that children must execute in the reading direction differ. 

While the reading errors in some children are caused by inappropriate saccade amplitudes, other children do not perform the correct sequence of staircase-like saccades in the reading direction. Instead, they perform searching eye movements in and against the reading direction and do not move the word segments to be read one after another into the area with sufficiently high visual acuity. The eye movements that children with dyslexia execute when reading text are often small. When the children try to read a sequence of words or word segments, they may realize that some words do not make sense. They assume that they did not correctly recognize words or word segments. Therefore, they execute one or more small eye movements opposite to the reading direction and try to recognize words or word segments that they did not recognize before. Having refixated these words or word segments, the readers execute a sequence of small eye movements in the reading direction until the gaze rests on the word or word segment from which the eye movements opposite to the reading direction started.

Readers may also realize that they cannot recognize word segments consisting of more than three, four, or five letters. Sometimes, readers compensate for this impairment by splitting the text into small word segments and read one segment after the other, resulting in a sequence of small reading eye movements in the reading direction. This strategy can improve reading performance, but reading performance may still remain below the age norm because the fixation times may still be too short, and the readers may still try to read more letters at a time than they can and/or may execute a saccade to the next word segment before the previous word segments have been recognized. Occasionally, the patients read letter-by-letter, resulting in a sequence of very small eye movements in the reading direction. Different inconvenient eye movement strategies constitute different kinds of dyslexia based on eye movements.

Whether eye movements are the cause of dyslexia cannot be proven by comparing the eye movements of children with dyslexia with the eye movements of typical readers. A person can read without error even if s/he executes eye movements that differ from those of typical readers. These include eye movements against the reading direction and/or eye movements whose amplitudes exceed the number of letters that can be recognized simultaneously. Such eye movements may be interspersed in a sequence of appropriate eye movements. It is only important that a sequence of appropriate eye movements is made, regardless of whether inappropriate eye movements occur between the appropriate eye movements.

The time it takes from the beginning of the fixation of a word segment to its pronunciation (verbal reaction time) can have a significant impact on whether a word segment is read correctly. If the verbal reaction time is shorter than the time it takes to retrieve the appropriate sequence of sounds from memory, reading errors will occur. Therefore, the verbal reaction time must be sufficiently extended. To achieve this, an acoustic signal was presented at a given time interval after the cursor moved to the next segment to be read. This time interval must be as long as the time intervals needed to recognize the pseudowords in the pseudoword experiment described above. However, the child will not begin to pronounce the word segment immediately after the acoustic signal; they will begin pronouncing the word segment at a variable time interval after the acoustic signal. In the pseudoword experiments [78,79,80,82], the computer measured and stored the time between the onset of the presentation of the marked pseudoword and the onset of the child’s correct pronunciation. When the computer guided eye movements, fixation location, fixation time, length of word segments, and verbal response time, there was an immediate 70% decrease in reading mistakes, whereas a control group reading without computer assistance did not improve its reading performance [76,77,78,79,80]. The children who read with computer assistance were not allowed to practice computer-assisted reading for hours or days because practice could have improved other influences on reading performance, such as visual attention. Then, it could not have been ruled out that the improved reading performance was at least partly due to improved visual attention. The error rate did not immediately drop to zero in children who read with computer assistance because the children did not always follow the computer’s instructions due to the lack of practice.

The results [76,77,78,79,80] show that appropriate eye movements (not eye movements that deviate from those of typical readers) and sufficiently long verbal reaction times are irreplaceable and necessary conditions for correct reading.

## 3. A New Look at Conditions and Causes

Based on what has been said so far, we can specify the concept of necessary and sufficient conditions for reading and the concept of cause. We do not concur with the attempts of philosophers of science to clarify the concept of cause [8,9,10,11,12,13,14,15,16,17], which is hardly applicable to the study of the causes of dyslexia. Here, we prefer an experimental approach rather than an approach based only on mathematical logic. We call conditions “necessary” if they are required for reading and cannot be replaced by other conditions. One such condition is the number of letters a reader tries to recognize simultaneously. Even when the fixation time was extended to 500 ms, many children in our studies were unable to recognize more than three, four, or five letters [78,79,80]. The condition that children are only allowed to try to recognize a limited number of letters is a necessary condition for reading a given number of letters simultaneously because this condition cannot be replaced by a different one, such as a longer fixation time or a longer verbal reaction time. A condition that must be established for error-free reading and that cannot be replaced by any other condition will be referred to as an “irreplaceable necessary condition”. Suppose we present a sequence of four-letter pseudowords for 250 ms each and find that these pseudowords cannot be recognized at this presentation time. Suppose that each pseudoword in a sequence of four-letter pseudowords can be recognized when the presentation time is increased to 400 ms, or when the presentation time is not increased from 250 ms but the letter size or contrast is increased. At least one of these conditions must be established for the pseudowords to be recognized. It does not matter which of the conditions is established. We call these conditions “replaceable necessary conditions”. If a person can read without error in a test, then all the conditions for error-free reading are established. If an irreplaceable necessary condition is removed, reading becomes impossible. If it is returned, the person will again be able to read without error. A replaceable condition is not necessary because it can be replaced by another (replaceable) condition.

Suppose a child is unable to read four-letter 3 mm-by-3 mm pseudowords that are colored red and presented for 300 ms on a white, yellow, or blue background. Suppose the child can read the pseudowords (1) if their size is increased to 5 mm-by-5 mm or (2) if the fixation time is increased to 400 ms. Suppose that the child cannot read the pseudowords (1) when their size is 5 mm-by-5 mm, or (2) when they are presented for 400 ms, but (3) when the red colored words are presented on a green background. This means that the pseudowords can be recognized if (1) the letters are 5 mm-by-5 mm, (2) the presentation time is 300 ms, and (3) when the pseudowords are presented on a white, yellow, or blue background; or, (1) if their size is reduced to 3 mm-by-3 mm, if (2) the presentation time is 400 ms, and (3) when the pseudowords are presented on a white, yellow, or blue background. Each of these conditions (size and presentation time) can be replaced by the other ones. The white, yellow, and blue backgrounds can be interchanged and the pseudoword can still be recognized. In the following definition, the interchangeable conditions “size vs. presentation time” are, e.g., elements of the set Λ1 (designated by the Greek letter lambda), the interchangeable backgrounds are, e.g., elements of a set Λ2, and so on. Each of these sets contains conditions that are interchangeable. It is not sufficient to distinguish solely between interchangeable conditions, it is also important to distinguish between different sets of interchangeable conditions because a condition in one set cannot be replaced by a condition in a different set. Only conditions that are elements of the same set can be exchanged; for example, size can be exchanged with presentation time and white, yellow, and blue backgrounds can be exchanged for one another.

The example above shows that there are different sets of interchangeable conditions. There is one set that contains the interchangeable conditions Λ1, …, Λp “size of letters” and “presentation time”. Another set of interchangeable conditions contains the conditions “white background”, “yellow background”, and “blue background”. For correct reading, the letters must have a certain size, or the presentation time must have a certain length, and the background must be white, yellow, or blue. No set of interchangeable sufficient conditions can be omitted. This means that each set (not an element of a set) of interchangeable sufficient conditions is necessary. At least one interchangeable condition from each set of interchangeable conditions must be satisfied (in the following, the terms “replaceable”, “exchangeable”, and “interchangeable” are used interchangeably). If correct reading is no longer possible if an irreplaceable condition is not met, this condition is an irreplaceable necessary condition. If correct reading is still possible even if this condition is not met, and if this condition cannot be replaced by another condition, then such a condition has no effect on reading performance and is therefore superfluous.

Not all necessary and interchangeable conditions are mentioned in the analysis of a person´s reading performance. Some are not mentioned because they are trivial, such as that the patients must keep their eyes open or that they have a functional visual system. The sets containing the interchangeable conditions may also be incomplete. In the example above, one may wonder what would happen if red letters were displayed on a brown background, which is not an element of the sets of interchangeable conditions. If the influence of a brown background has not yet been investigated, nothing is known about its influence on reading performance. It is a matter of research to investigate the influence of a brown background and to increase our knowledge about irreplaceable necessary and replaceable conditions.

A detailed look at the reading experiments shows that the earlier distinction between necessary and sufficient conditions, which is based only on the reasoning of mathematical logic, is not useful and must be replaced by more sophisticated definitions based on experimental methodology. Since a replaceable condition cannot be a necessary condition, but only a sufficient condition, we call exchangeable conditions “sufficient exchangeable conditions”. However, the concept of “sufficient condition” used in Definition I is not identical to this concept based on mathematical logic [14,15,16,17]. Here, we extend a previous view [18]:

**Definition** **1.** *Let **Δ** be the set of all conditions under which a person P can read flawlessly. Let **Γ** be a subset of **Δ** that contains only the conditions N1, …, Ni…, Nk, and let Λ1, …, Λi, …, Λp be different subsets of **Δ**. Λ1 contains only the elements (conditions) H1, …, Hq. Λi contains only the elements K1, …, Kr, and Λq contains only the elements M1, …, Mu.*

*(1)*  *Then, the elements of **Γ** (i.e., the conditions N1, …, Ni, …, Nk) are irreplaceable necessary conditions if and only if the following is valid for each element of **Γ** and only for the elements of **Γ**.*
*Correct reading is no longer possible when at least one element of **Γ** is missing and at least one element of each set Λ1, …, Λi, …, Λp is present. This means that **Γ** contains all irreplaceable necessary conditions and only irreplaceable necessary conditions.*
*(2)*  *An element of a set Λi out of the sets Λ1, …,* *Λi, …, Λp* *is a replaceable sufficient condition (but not an irreplaceable necessary condition) for correct reading if correct reading is possible if and only if:*
  *(i)*  *Each subset Λ1, …, Λi, …, Λp contains more than one element (condition).*
  *(ii)*  *At least one element (condition) of each set Λi out of the sets Λ1, …, Λi, …, Λp is met.*
  *(iii)*  *All elements of **Γ** are met.*
  *(iv)*  *Correct reading is no longer possible if none of the conditions that are elements of a set Λi out of the sets Λ1, …, Λi, …, Λp is met, even if all elements of **Γ** are met.*
*(3)*  *A set Λi (not an element!) out of the sets Λ1, …, Λi, …, Λp is necessary for correct reading if and only if correct reading is only possible if at least one element of this set is met.*

The concept of causation can then be defined as follows:

**Definition** **2.**

*(1)*  *It is an irreplaceable necessary cause of a reading impairment if at least one element of **Γ** is not met (this corresponds to item (1) of Definition 1).*
*(2)*  *It is a replaceable sufficient cause of a reading impairment if none of the elements of at least one set Λi out of the sets Λ1, …, Λi, …, Λp is met (this corresponds to items (2) and (3) of Definition 1).*
*(3)*  *If a condition that is not an irreplaceable necessary condition is not met, or if a condition that is not a replaceable sufficient condition is not met, this cannot be the cause of a reading impairment.*

This definition assumes that a person either can or cannot read correctly. Many children can read but their reading performance is just below the age norm and the question is how to improve reading performance. In such cases, the definition can be modified by replacing the term “correct reading” with the term “reading performance at level L”. Level L may, for example, be the rate of reading errors and may correspond to the age norm. If a child’s reading performance is below level L, it must be investigated which conditions are missing so that level L is not achieved, and how the conditions can be established so that the person can read at level L. This conceptual framework allows us to describe in detail all different kinds of missing conditions that cause dyslexia.

Now, we can describe the conditions of the hemianopic reading disorder in terms of Definitions 1 and 2 as follows: the absence of an expansion of the visual field in the foveal and parafoveal area after visual field training, and the absence of compensatory eye movements are replaceable conditions for the occurrence of a hemianopic reading disorder.

The results of the pseudoword experiments can be described as follows:

(1) Too short a fixation interval and trying to recognize too many letters at the same time are replaceable conditions for recognizing at least 95% of a sequence of 20 pseudowords in some children.
(2) Too short a fixation interval is an irreplaceable necessary condition for recognizing at least 95% of a sequence of 20 pseudowords in some children.
(3) Trying to recognize too many letters at the same time is an irreplaceable necessary condition for recognizing at least 95% of a sequence of 20 pseudowords in some children.
(4) A given verbal reaction time is an irreplaceable necessary condition for correctly pronouncing the pseudowords.
(5) Searching eye movements are irreplaceable necessary causes of dyslexia.
(6) Hypermetric saccades in the reading direction that exceed the number of letters that can be recognized simultaneously and that are not followed by a correction saccade opposite to the reading direction are an irreplaceable necessary cause of dyslexia.
(7) Too short verbal reaction times are irreplaceable necessary causes of dyslexia.
(8) Searching eye movements are neither necessary nor sufficient cause of dyslexia when they are interspersed in a sequence of appropriate reading eye movements. The same is true for single saccades opposite to the reading direction.
(9) A sequence of small saccades in the reading direction can be a sufficient cause of slow reading but is not yet sufficient for correct reading.

There are certainly many other conditions that are not mentioned in the analysis of a person´s reading performance because they are trivial. Others may not be mentioned because they still need to be studied. If experimental studies find features and impairments associated with dyslexia but meet item (3) in Definition 2, these are coexisting features or impairments that are not causally related to dyslexia.

## 4. High-Impact Rapid Therapy That Compensates for the Causes of Dyslexia

So far, the goal has been to identify the conditions and causes for reading. Dyslexia was caused by the absence of one or more of these conditions. These causes, which included inappropriate eye movements, inappropriate fixation location, too short fixation times, trying to recognize too many letters simultaneously, and premature pronunciation of the word segments to be read, were not the same in different children. Therefore, the therapy had to be adapted to the causes of dyslexia of each individual child. Since children needed different fixation times, these had to be prolonged differently in different children. As the number of letters that children were able to recognize simultaneously also differed considerably, the length of segments into which a text had to be split differed among children. The verbal reaction time needed also differed among children when reading aloud so the computer dictated different verbal reaction times for different children during therapy. Since the amplitude of the saccades in the reading direction had to be adapted to the different lengths of the word segments that different children could recognize simultaneously, the computer instructed different children to execute saccades of different amplitudes.

Successful therapy depends on identifying the conditions and causes of dyslexia. To significantly improve the ability to read a text without the help of a computer, the appropriate eye movements, the appropriate fixation location, the appropriate fixation time, the number of letters to be recognized simultaneously, and the appropriate verbal response time were first demonstrated on a computer. The children then practiced the appropriate reading strategy for approximately 25 min with the computer. They were then asked to transfer this strategy to reading a normal text in a book without the help of a computer. In four studies [76,77,78,79,80] including 356 children with dyslexia, the number of errors was reduced to about one-third within this short period of time. In all of our studies, the dramatic improvement in reading performance occurred in less than half an hour, whereas traditional therapy approaches that often take months and sometimes years are unspecific and have limited success.

There can be many influences on reading performance that cannot be controlled by the therapist within a period of a few weeks. Allowing for uncontrolled variables is contrary to the essential requirements of a scientific study. All potential variables affecting reading performance can be controlled only if the reading improvement is achieved in a single therapy session. In this type of therapy, impairments in the abilities required for reading are compensated for by increasing the fixation times and the verbal reaction times, limiting the number of letters that can be recognized simultaneously and learning the appropriate eye movements for reading.

In addition to this compensatory therapy, there is also a type of therapy that attempts to improve poor reading performance through training, i.e., to improve the ability to recognize several letters simultaneously, to shorten the fixation time required to recognize a sequence of letters simultaneously, and to shorten the verbal reaction time required for reading. It is possible to perform such a therapy using the computer program described above. However, a therapy aimed at improving impairments may take months or even years, so that influences on reading performance outside the therapy cannot be controlled. There are also biological limitations that cannot be overcome, so that no therapy can improve all abilities that are a prerequisite for adequate reading.

When a reader tries to read a text fixating the word in the middle and trying to simultaneously recognize all letters in the word, reading mistakes are inevitable when words contain more letters than the reader can recognize at a time. When words are long, letters at the beginning and the end of the word may be outside the area of sufficient visual acuity. The whole word cannot be seen, and unseen letters must be guessed. Therefore, reading eye movements should not jump from one word to the next. Instead, the text must be split into segments that do not exceed the width of the area of sufficiently high visual acuity. One approach is to split the text into syllables. The ability to split a text into syllables and sounds is one feature of so-called “conscious awareness” [65,66,67,68,69,70,71,72,73,74,75]. Splitting the text into syllables improved the reading ability of poor readers [116]. However, syllables may contain more letters than a reader can simultaneously recognize within a fixation interval. We have demonstrated that the ability to simultaneously process a string of letters differs among children, depends on the length of the fixation interval, and is limited to five letters or less in most children with dyslexia. Only a few children were able to simultaneously recognize more than five letters even if the fixation time was prolonged by up to 500 ms. If a reader tries to simultaneously recognize more letters than they can, reading mistakes will occur. If syllables contain fewer letters than a reader can recognize simultaneously, the reader will split the text into unnecessarily small segments, which makes reading slow and may impair understanding. Therefore, it is not a good reading strategy to divide the text into syllables, regardless of a reader’s ability to simultaneously recognize a string of letters. Instead, the text should be divided into segments whose length are tailored to the reader´s ability to simultaneously recognize a string of letters. We tested how many letters children with dyslexia can read simultaneously depending on the length of the fixation interval using pseudowords. Reading pseudowords differs from reading natural words because they can only be recognized when every letter is identified, whereas natural words can already be recognized if only some letters are identified and the remaining letters are guessed. Therefore, natural words are inappropriate to investigate whether a reader can recognize all the letters that make up a word. If it is known how many letters a reader can recognize simultaneously within a given fixation interval, the lengths of the segments that should be recognized simultaneously and which the computer indicates with a red and a green cursor could be tailored to the reader´s ability. Since some readers may be able to recognize natural words that are longer than pseudowords, we also tested whether the segments in natural words can be longer than in pseudowords when reading a text. In our studies and in clinical routines, only some children could read flawlessly when natural words were no more than one letter longer than pseudowords. In a study by Müller et al. [116], who used a syllable segmentation approach in a reading therapy in which German-speaking children participated, the effect size was g = 0.8. In our studies, the therapy reached an effect size (Hedges g) of up to 2.03 in a free-reading test 30 min after therapy [76,77,78,79]. When children were reading with the help of a computer, the effect size even reached g = 2.137 [80]. This demonstrates that the requirement to split the text into segments is correct. The number of characters the segments should contain must, however, be tested beforehand.

Castles and Coltheart [117] argued that no causal relationship between phonological awareness and the ability to learn to read has been proven because the studies on conscious awareness do not fulfill their requirements for a causal relationship. Hulme et al. [118] argued that these requirements are too narrow and assume that there is, indeed, a causal link. There is no doubt that reading presupposes knowledge of the correspondence between graphemes and phonemes and between a sequence of graphemes and a sequence of phonemes. Even if poor performance in abilities that belong to conscious awareness predicts failure in learning to read, this is not evidence of a causal link between reduced conscious awareness and dyslexia. According to Definition 1, such a causal link can only be proven when reading ability improves significantly after it has been tested for each component of conscious awareness that reading performance improves significantly after this component has been normalized or compensated and all other possible influences on reading performance have been excluded. It must, e.g., be excluded that the child´s motivation to read, the ability to focus visual attention, the ability to simultaneously recognize a sequence of letters, to divide the text into appropriate word segments, to fixate on the word segments to be read for a sufficiently long time interval, and not to pronounce the word segments too early, improved during therapy. If these aspects improved, it could not be determined which one caused dyslexia. All these factors remained uncontrolled in the studies on the impact of conscious awareness on reading performance. When a therapy takes weeks or months, there are also many other influences on reading performance that remain uncontrolled. Thus, we fully agree with the argument of Castles and Coltheart [117] that a causal link between phonological awareness and success in learning to read has never been proven. In our studies, all possible causes were studied individually and the therapy was completed within less than 30 min so that all influences could be controlled. In one study [80], the computer dictated a new reading strategy that compensated for impaired abilities necessary for reading so that no training was applied that could have changed any uncontrolled abilities that could have influenced the reading performance.

Some children exchange the sounds that belong to p and q, b and d, and m and n but have no problems associating all other letters with the correct sounds. In our clinical routine, this is a problem for only a minority of children with reading problems and it is by no means a general feature of dyslexia. All the children in our studies knew all the letters. However, children with dyslexia may need more time than typical readers to retrieve the correct sequence of sounds that belong to a sequence of letters from memory. In our studies [78,79,80], the average verbal reaction time was between 1316 ms (SD = 712 ms) and 1670 ms (SD = 641 ms). A 12-year-old boy (not included in the studies) whom we saw in clinical routine was an extreme example of a prolonged verbal reaction time. He even took between 9 and 11 s to retrieve the correct sounds from memory in the pseudoword test. After this long verbal response time, he was able to correctly recognize all four-letter pseudowords of a series of 20 pseudowords. In languages with a low grapheme–phoneme correspondence, such as English and French, it is more difficult to learn this correspondence and to retrieve it from memory than in languages with a high grapheme–phoneme correspondence such as German, Italian, and Spanish. Our studies show that children may suffer from severe dyslexia even if they know all the associations between graphemes and phonemes. This demonstrates that difficulties associating phonemes with graphemes cannot be a general cause of dyslexia but may only increase verbal reaction time. Our studies demonstrated that reading problems due to the need for a prolonged verbal reaction time only occur when the children do not adhere to the verbal reaction time that they need and start pronouncing too early. The question of whether a prolonged verbal reaction time due to difficulties associating phonemes rapidly with graphemes is a cause of dyslexia must always be answered regarding the verbal reaction time needed, including the duration of the fixation interval that readers require.

Some researchers argued that DD is caused by a lack of visual attention [30,31,32,33,34,35,36,37,38,39,40]. Our experiments show that this is not the case. Referring to a precise concept of attention [18], two features of visual attention can be distinguished. That attention is focused on a location means that all effort is focused on a retinal area to visually process a stimulus that is displayed or that is expected to appear there. The pseudoword experiments demonstrate that at least 95% of 20 pseudowords were recognized correctly when the children had enough time to focus their attention and when the fixation time was sufficiently prolonged. Before the pseudoword was displayed, the children directed their gaze for some seconds to the location where they expected the pseudoword. This allowed for sufficient time to focus attention. The number of letters a reader could recognize at a time depended on the fixation time but not on the time the reader could focus their attention, i.e., the period from the beginning of fixation of a fixation mark waiting for the pseudoword to appear at this location and the presentation of the pseudoword. However, the number of letters that children were able to recognize simultaneously differed among children even if the fixation time was extended by up to 500 ms. Even then, some children were only able to recognize three letters, some could recognize four letters, others could recognize five letters, and few children could simultaneously recognize six letters. This shows that the number of letters that could be recognized simultaneously did not depend on a limited ability to focus attention but on the fixation time and on the individual capacity to simultaneously recognize several letters independent of the fixation time.

The ability to extend the field of attention is another feature of visual attention. It has been shown that this is a cerebral capacity of its own that may be impaired independently of other cerebral capacities [119,120]. If dyslexia was caused by the reader´s inability to extend their visual field of attention, one would expect the reader to miss letters at the beginning and the end of words. The pseudoword experiments demonstrated that this was not the case [78,79,80,82]. Reading errors of all kinds occurred at all locations in pseudowords, and letters at the beginning and at the end of pseudowords were not misread or overlooked more often than letters at other positions in the pseudowords. In conclusion, our studies [76,77,78,79,80,82] demonstrated that the reading problems in the children who participated in our experiments were not caused by a lack of visual attention.

The finding that there was no difference in the rate of reading mistakes regardless of whether a letter was flanked by other letters on both sides or whether it was only flanked by a letter on one side as was the case with the last letter of the pseudowords does not agree with the assumption that visual crowding [19,20,21,22,23,24,25,26,27,28,29,30] is the cause of DD. If DD were caused by visual crowding, the second or the third last letter of a pseudoword flanked on both sides by other letters would be misread or overlooked significantly more often than the last letter not flanked by letters on both sides. This was not the case [78,79,80,82].

Since the fixation point was in the middle of the pseudowords, the first and the last letters in a five- or six-letter pseudoword were further in the periphery than the letter at the fixation point that was in the fovea. The first letter that was further in the periphery was less frequently misread than the letter in the fovea and there was no significant difference between the frequency of reading mistakes of the first, the second, or the third letter to the right of the fovea, which were at different distances from the fovea. Thus, there was no indication of a decline in reading performance for letters that were two or three letters away from the fovea [78,79,80,82].

DD has also been explained by the magnocellular theory of DD [54,56,59,60]. The theory assumes that DD is caused by an impaired function of the magnocellular visual pathway, resulting in impaired saccadic suppression, poor binocular convergence, and poor control of reading saccades. Retinal ganglion cells are composed of magnocells, parvocells, and koniocells (for review, see Ref. [81]). A total of 10–20% of the retinal ganglion cells are magnocells and 80% are parvocells. The ratio of parvocells increases toward the periphery of the visual field. Parvocells have higher visual acuity and convey color information, whereas magnocells conduct information faster than parvocells, convey information about fast-moving visual stimuli, are more activated by high temporal contrast, and can detect flicker better than koniocells, but magnocells have poor visual acuity. Magnocells send fibers to the two ventral layers of the lateral geniculate body, whereas parvocells project to the remaining four layers. The assumption that DD is due to an impaired function of magnocells has been criticized by different authors [121,122,123,124,125]. Previous studies demonstrated that detection and recognition of visual stimuli [126,127,128,129,130,131,132] and visual acuity [133,134,135,136,137,138] increase with longer fixation times. This is a result of the temporal summation of visual stimuli, which is pronounced in Areas V1, V2, and V3 of the occipital cortex [139]. Our results demonstrate that children with dyslexia need longer fixation times to generate sufficient temporal summation in occipital neuronal networks to detect and recognize all letters of pseudowords [78,79,80,82]. This concurs with the magnocell theory of DD insofar as it demonstrates that DD is a result of impaired sensory processing. We cannot, however, distinguish between the contribution of different cell types in the visual system. Both parvocells and magnocells may contribute to temporal summation in occipital neural networks. The finding that children with dyslexia can execute appropriate reading eye movements without training when the eyes are guided by the computer [80] and without the help of a computer after having learned to execute appropriate eye movements within 30 min [76,77,78,79] demonstrates that the eye movement system of children with DD is not functionally impaired. This contradicts theories that assume that dyslexia is caused by impaired control of reading eye movements [51,54,55,56,59,60,61,62,63,64]. A therapy aimed at improving reading performance in children with dyslexia using eye movement training under non-reading conditions [140] included a therapy group of only 10 children who performed daily eye movement training at home and a control group of 11 children who received no alternative training. The effect size and the duration of the training was not reported but was presumably about 50 days according to a later-reported single-case report [141]. In contrast, our studies involved 356 children with dyslexia. The therapy group reached an effect size up to g = 2.137 [76,77,78,79,80].

It has also never been proven that impaired auditory discrimination causes dyslexia, as proposed by some authors [41,42,43,44,45]. Auditory discrimination training that rested on this assumption had no effect on reading performance [142,143,144]. The highest effect size that Galuschka et al. [145] found in their survey of different kinds of reading therapies was a small effect size of g = 0.322 for therapies consisting of phonic instructions in spelling lasting at least 12 weeks. In a survey by Ise et al. [146], only five out of sixteen therapy sessions yielded an effect size of between g = 0.6 and g = 1.18. These therapies lasted between 6 weeks and 10 months. All other therapies were either ineffective or had only a minimal effect. As mentioned above, it is not possible to control all influences on the improvement of reading performance within such a long time when the children are not controlled under standardized laboratory conditions. In our studies, the diagnosis and therapy were completed in one session in which all influences were controlled. The therapy yielded an effect size of g = 2.137 when children read with the help of a computer but without any reading training and an effect size of up to g = 2.03 when reading without the help of a computer after less than 30 min of reading therapy.

## 5. Conclusions

The term DD covers various types of reading disorders that must be differentiated diagnostically. Since the various reading problems differ according to their causes, identifying them must be part of the diagnostic process. The conditions required for correct reading and their lack that causes dyslexia cannot be adequately described by the terms “necessary conditions”, “sufficient conditions”, and “cause”, as they are used intuitively in experimental psychology and have been made explicit in the philosophy of science. Methods for revealing the conditions that must be fulfilled for correct reading and for identifying the causes of different types of dyslexia have been described and their application has been demonstrated. To explain the results of the application of these methods and the various causal relationships, a sufficiently detailed conceptual framework has been proposed. Within this framework, the concepts of “necessary conditions”, “sufficient conditions”, and “causation” have been replaced by more convenient concepts that allow us to describe in great detail the conditions required for correct reading and the causes of different types of dyslexia. Dyslexia therapy based on such a differentiated diagnosis and tailored to each child´s reading problems has proven to be immediately highly effective.

## Data Availability

No new data were created or analyzed in this study. Data sharing is not applicable to this article.
